# Supplementary material for: Quantifying the critical thickness of electron hybridization in spintronics materials
Source: Nat Commun. 2017 Jul 17;8:16051. doi: 10.1038/ncomms16051 (PMC5520016; doi:10.1038/ncomms16051)
Supplement: Supplementary Information [file ncomms16051-s1.pdf]

File Name: Supplementary Information
Description: Supplementary Figures, Supplementary Tables, Supplementary Notes and Supplementary References

File Name: Peer Review File
Description:

Supplementary Figures:

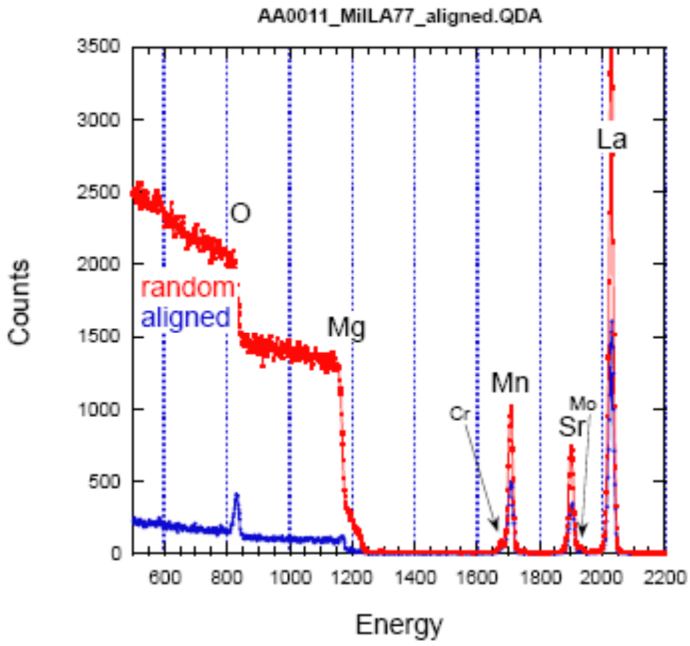

Supplementary Figure 1. $La_{0.67}Sr_{0.33}MnO_3$ Rutherford Backscattering. Rutherford back scattering measurements on LSMO 100 u.c. thick film, showing the alignment of atomic channels in a crystalline, highly ordered lattice.

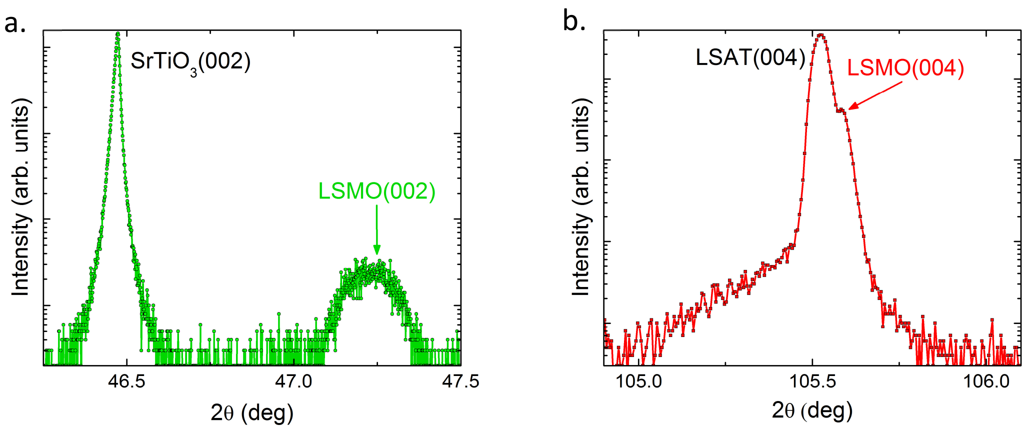

Supplementary Figure 2. $La_{0.67}Sr_{0.33}MnO_3$ XRD two-theta omega scans. a. 2theta-omega XRD measurements for LSMO 100 uc film deposited on STO(001) substrate. **b.** 2theta-omega XRD measurement for LSMO 100 uc film deposited on LSAT (001) substrate.

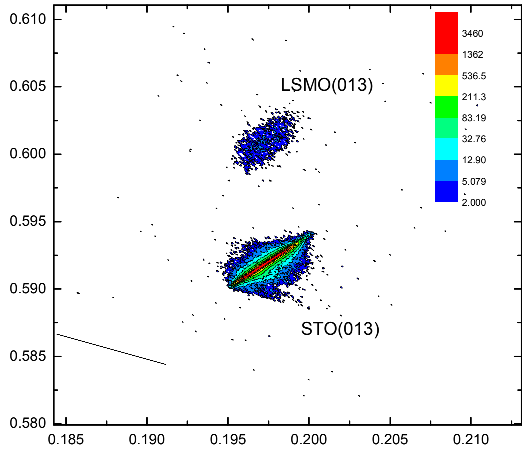

Supplementary Figure 3. $La_{0.67}Sr_{0.33}MnO_3$ XRD Reciprocal space map. Reciprocal space map around the asymmetric diffraction peak STO(013). The same position of substrate and film peaks in x axis is due to completely in-plane strained film.

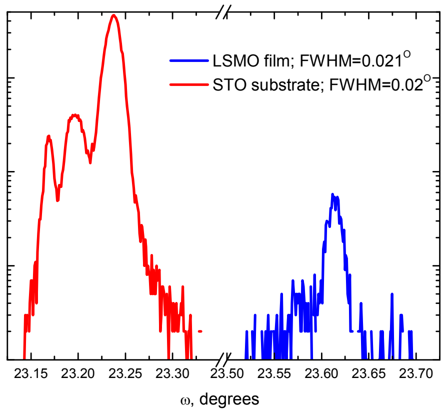

Supplementary Figure 4. $La_{0.67}Sr_{0.33}MnO_3$ XRD rocking curves. XRD Rocking Curves for 100 u.c. thick $La_{0.65}Sr_{0.35}MnO_3$ film (blue) and substrate (red) for film deposited on STO(001).

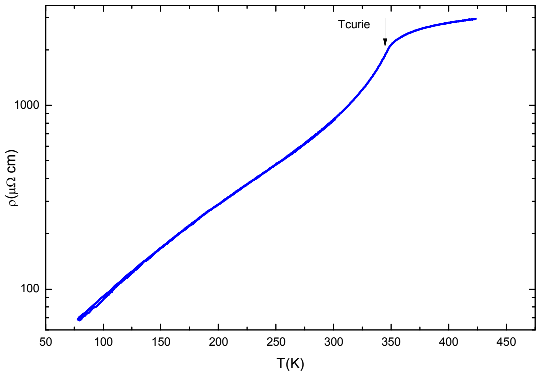

Supplementary Figure 5. $La_{0.67}Sr_{0.33}MnO_3$ Resistivity. LSMO 100 uc thin film resistivity vs temperature. Film deposited on STO(001) substrate. The arrow indicates the Curie temperature value.

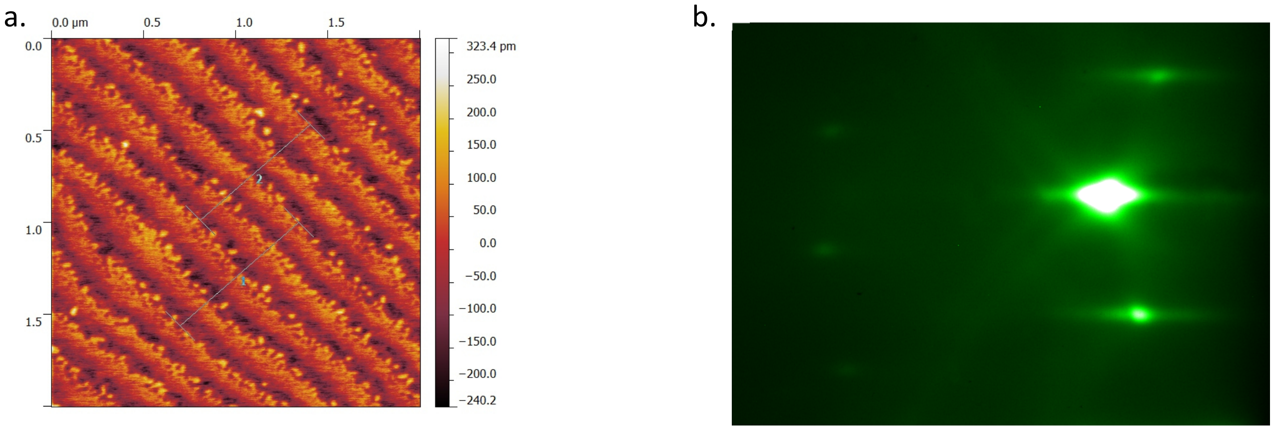

Supplementary Figure 6. $La_{0.67}Sr_{0.33}MnO_3$ AFM and RHEED. a. AFM and **b.** RHEED images of typical LSMO films deposited on STO(001) substrate.

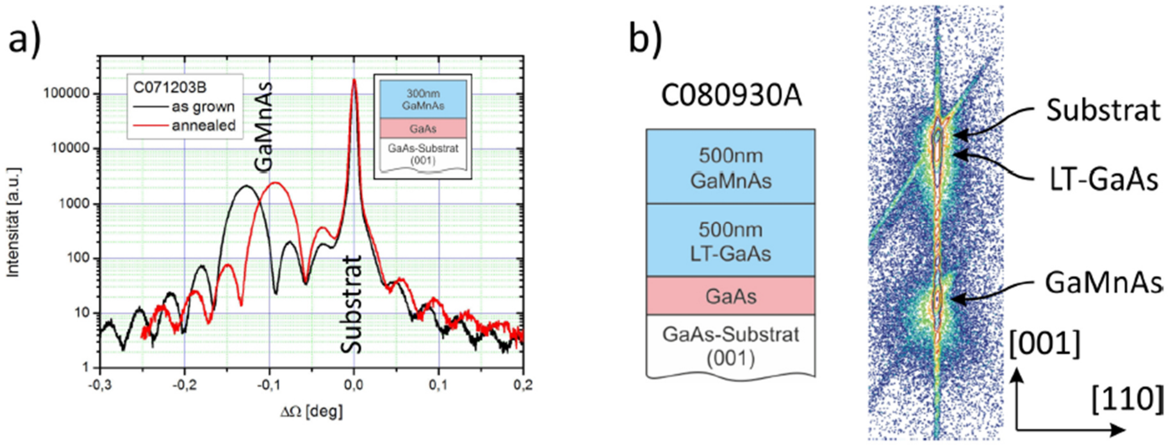

Supplementary Figure 7. (Ga,Mn)As growth and characterization. a. XRD spectrum (rocking curve) of a 300 nm thick (Ga,Mn)As film grown on LT-GaAs/ GaAs(001). The black curve shows the as grown data, the red curve is recorded after annealing. **b.** reciprocal lattice map of 500 nm (Ga,Mn)As grown on LT-GaAs/GaAs(001).

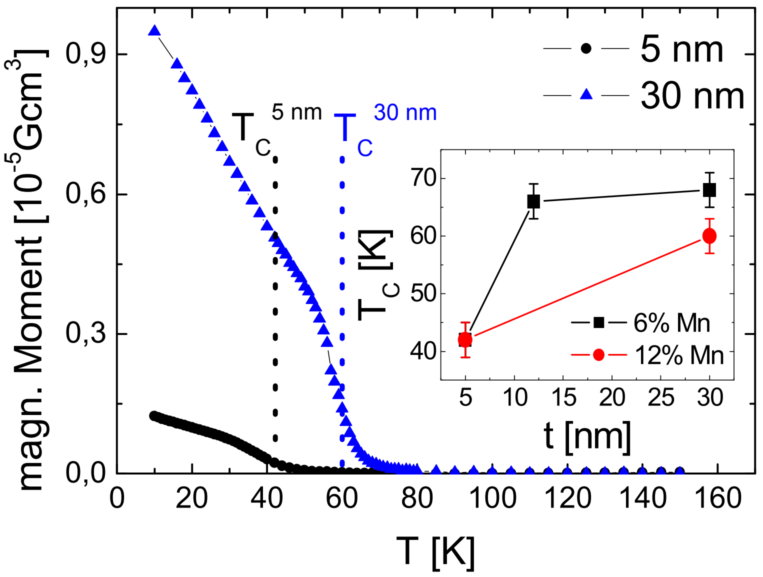

Supplementary Figure 8. (Ga,Mn)As magnetism. SQUID data of as grown thin film (Ga,Mn)As samples with different Mn concentrations. The blue curve shows the temperature dependent magnetization of a 30 nm thick film (x=12%), the black curve shows data for a 5 nm thick film with the same Mn concentration. The inset shows the evolution of the Curie temperature determined by the inflection point of the SQUID data as a function of film thickness for two Mn concentrations (6% and 12%), in the case as-grown films. The error bars in the inset are determined by the uncertainty on the fitting parameters.

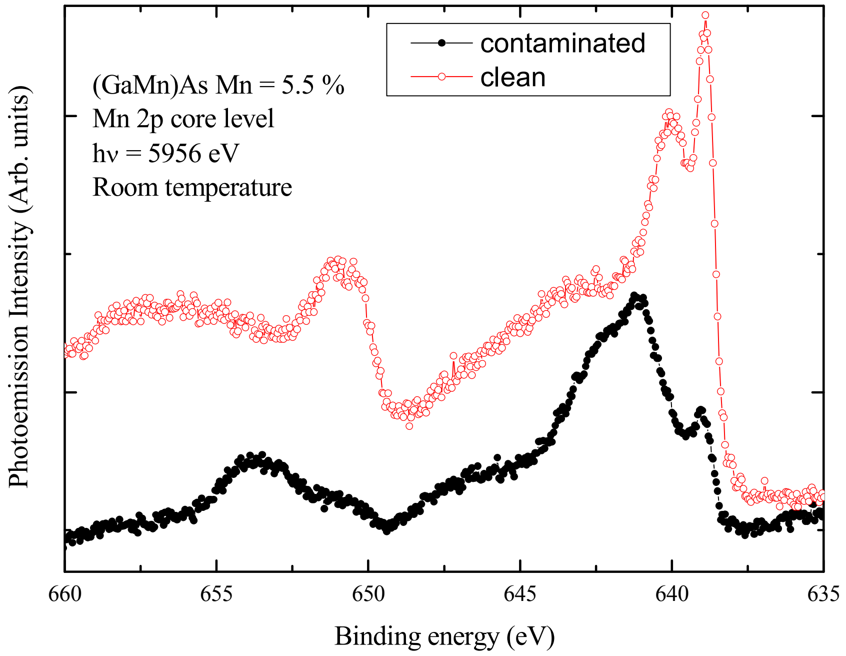

Supplementary Figure 9. Contamination in HAXPES. HAXPES Mn 2*p* PES of (Ga,Mn)As (5.5% Mn) measured at room temperature for clean (red open circles) and contaminated sample (black filled circles).

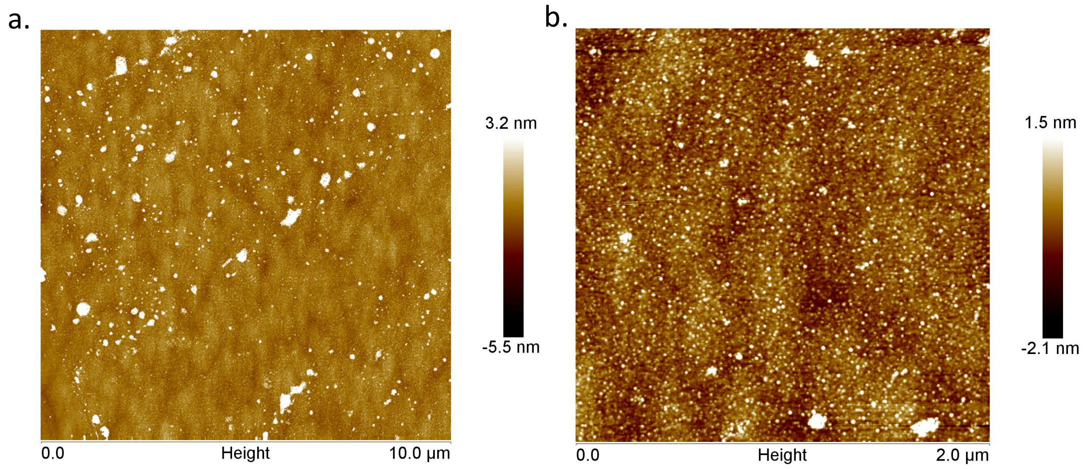

Supplementary Figure 10. (Ga,Mn)As surface roughness. a. 10 x 10 µm^2 and **b.** 2x2 µm^2 AFM images of untreated sample.

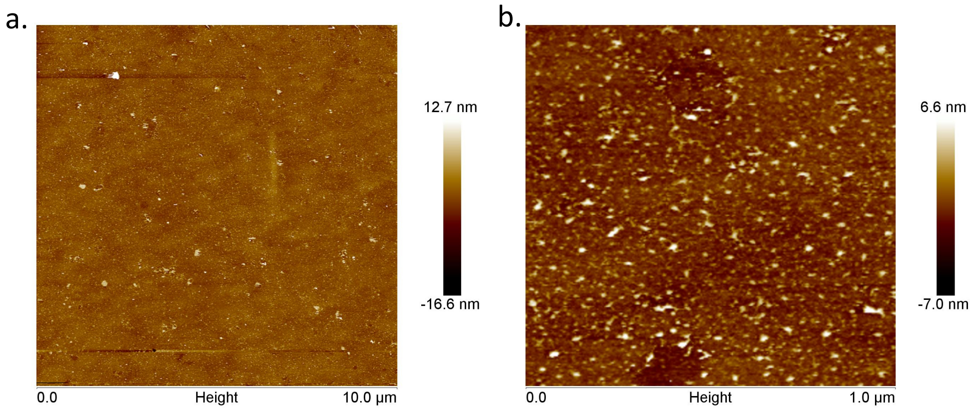

Supplementary Figure 11. Etched (Ga,Mn)As surface roughness. a. 10 x 10 µm^2 and **b.** 2x2 µm^2 AFM images of samples after chemical etching.

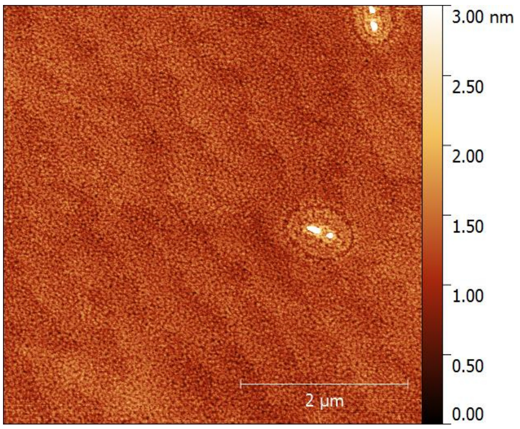

Supplementary Figure 12. $La_{0.67}Sr_{0.33}MnO_3$ surface roughness. 5x5 µm^2 AFM image of LSMO thin film. The scale bar on the bottom right corner spans a width of 2 µm.

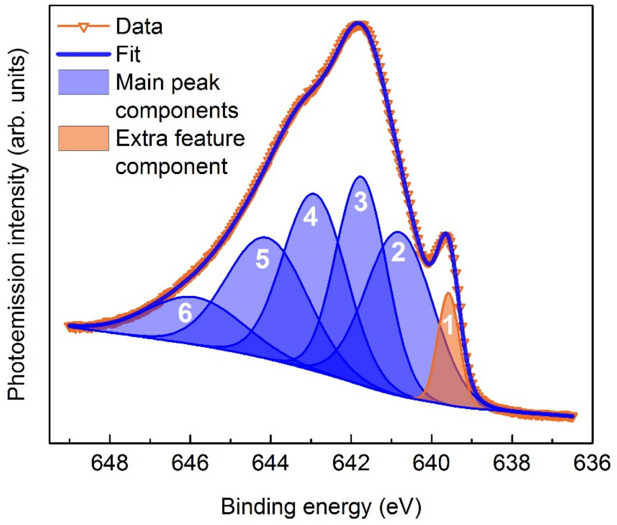

Supplementary Figure 13. Fitting procedure. Typical peak fitting result. In this case, LSMO Mn 2p 3/2 line at 5940eV photon energy is analyzed (T=200K). The open triangles show the data, and the solid line the result of the fit. The shaded curves from 2 to 6 are the Gaussian components of the main peak, while the shaded curve 1 is the Gaussian component used to fit the well-screened satellite.

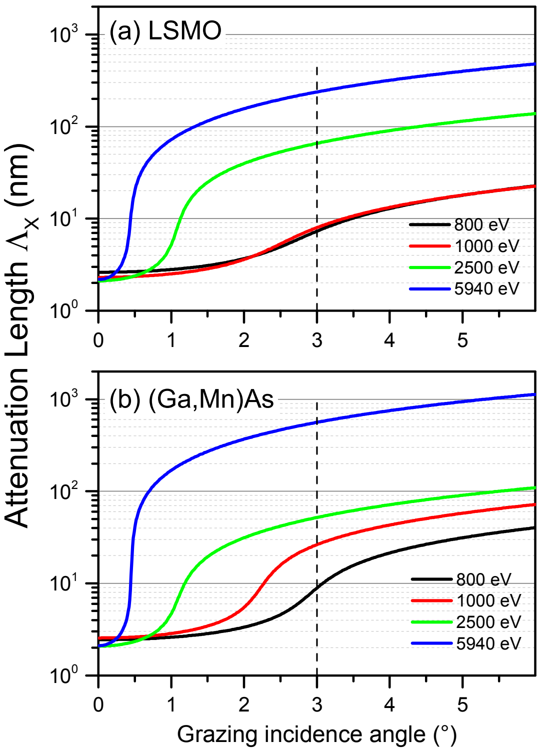

Supplementary Figure 14. Grazing incidence X-ray penetration depth. Dependence of the x-ray attenuation length λ_X on the grazing incidence angle for the photon energy of interest. The dashed line indicates the condition adopted in our experiment, namely 3 degrees of grazing incidence.

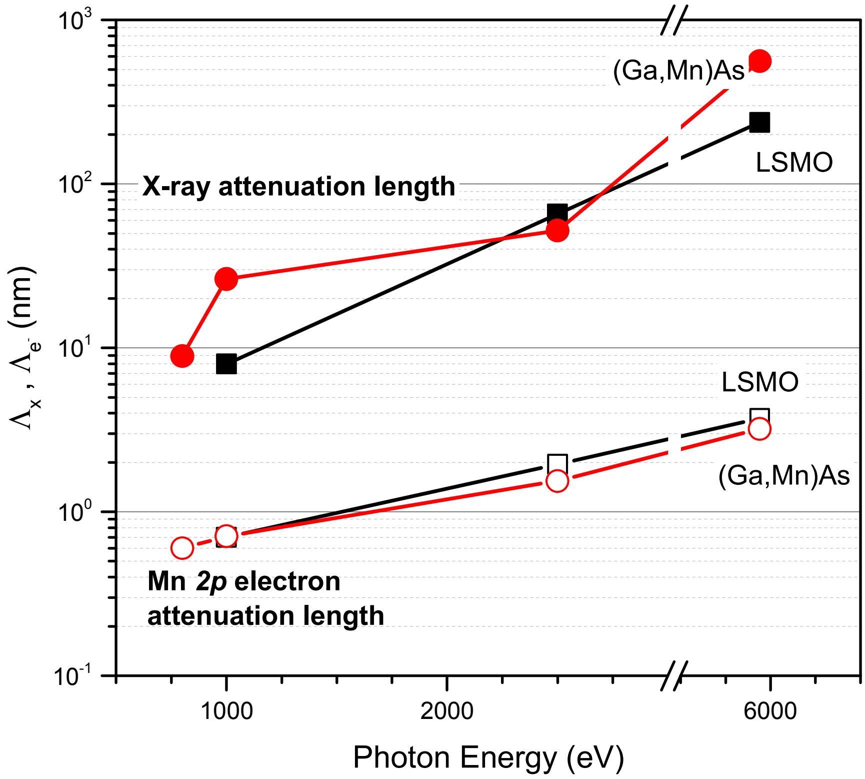

Supplementary Figure 15. X-ray penetration depth versus electron escape depth. Comparison of the attenuation lengths for the incident x-rays (λ_X) and the Mn 2p photoelectrons (λ_{e^-}) for each excitation energy adopted in the experiment.

Supplementary Tables

% Mn	T_g (°C)	d_{GaMnAs} (nm)	T_C (K)	σ_{300K} (Ω^{-1} cm^{-1})	σ_{5K} (Ω^{-1} cm^{-1})
1 ± 0.1	269	50	< 4	93.5	0.41
5 ± 0.2	230	35	60±2	152	112
13 ± 0.7	190	18	80 ± 2	209.2	189.8

Supplementary Table 1. (Ga,Mn)As growth parameters. Parameters of GaMnAs films grown on GaAs(001), with Mn percentage, growth temperature *Tg*, thickness *d*, Curie Temperature *Tc*, resistivity at 300 K and 5K.

Ph. En. (eV)	Peak	Pos. (eV)	Amplitude	Gaus. FWHM (eV)	Lor. FWHM (eV)
800	1	638.99	1.00	0.52	0.20
	2	639.68	2.26	1.42	0
	3	640.60	3.38	1.61	0
	4	641.55	7.56	2.55	0
	5	643.31	6.38	3.24	0
	6	646.28	7.60	4.75	0
1000	1	638.80	1	0.57	0.15
	2	639.50	1.11	1.604	0
	3	640.36	1.28	1.77	0
	4	641.38	1.45	2.59	0
	5	643.24	1.63	3.13	0
	6	645.89	1.78	4.09	0
2500	1	639.10	1.00	0.52	0.27
	2	639.91	2.18	1.53	0
	3	640.44	2.47	1.96	0
	4	641.39	4.16	2.59	0
	5	643.34	3.79	3.19	0
	6	645.84	3.52	4.33	0
5940	1	639.13	1.00	0.52	0.25
	2	639.95	0.98	1.32	0
	3	640.64	1.00	1.65	0
	4	641.79	1.00	2.39	0
	5	643.72	1.01	2.80	0
	6	646.00	1.01	3.70	0

Supplementary Table 2. (Ga,Mn)As Mn2p 3/2 fit parameters. Fitting parameters for the Mn 2p3/2 spectra of the $Ga_{0.87}Mn_{0.13}As$ sample. Peak 1 identifies the well-screened satellite.

Ph. en. (eV)	Peak	Pos. (eV)	Amplitude Gaus.	FWHM (eV)
1000	1	639.43	1	0.66
	2	641.37	65	1.50
	3	640.38	53	1.73
	4	642.42	73	1.73
	5	643.77	52	2.21
	6	645.57	32	3.07
2500	1	640.01	1	0.57
	2	641.83	9.3	1.48
	3	640.75	6.1	1.47
	4	642.83	14	1.95
	5	644.21	10	2.31
	6	645.85	6.5	3.08
5940	1	639.58	1	0.63
	2	641.75	4.4	1.48
	3	640.80	4.5	1.95
	4	642.91	4.7	1.85
	5	644.09	4.0	2.36
	6	645.86	1.9	2.81

Supplementary Table 3. $La_{0.67}Sr_{0.33}MnO_3$ Mn2p 3/2 fit parameters. Fitting parameters for the Mn 2p3/2 spectra measured at T = 200 K on the $La_{0.66}Sr_{0.33}MnO_3$/LSAT sample. Peak 1 identifies the well-screened satellite.

	**LSMO**		**(Ga,Mn)As**	
**Photon Energy (eV)**	**λ_{ph} (nm)**	**λ_{e^-} (Mn 2p) (nm)**	**λ_{ph} (nm)**	**λ_{e^-} (Mn 2p) (nm)**
**800**	-	-	8.90	0.60
**1000**	7.98	0.70	26.33	0.71
**2500**	65.69	1.95	51.95	1.54
**5940**	237.35	3.71	561.77	3.20

Supplementary Table 4. Attenuation length data. Summary of the data plotted in Supplementary Figure 15.

Supplementary Note 1: Structural and magnetic characterization of LSMO thin films

The films of $La_{1-x}Sr_xMnO_3$ (LSMO, $0.3 \le x \le 0.35$) were deposited on the substrate by reactive Molecular Beam Epitaxy (MBE) in atmosphere of pure ozone. The alternated shuttering method, developed by Schlom and co-workers was used for the film growth [1]. Due to very small mismatch in lattice parameters (≤ 1%) with optimally doped LSMO film, STO(001) and LSAT(001) substrates were chosen. LSMO films up to 40 nm (100 u.c.) of thickness were grown in ozone pressure ($5x10^{-7}$mBar) at substrate temperature around 750°C. Note that fine adjustment of the temperature depends on the substrate and is obtained by optimizing the structural characteristics of the grown film. Stoichiometry of the perovskite structure was followed in-situ and in real-time during film growth (and adjusted if necessary) by means of reflection high energy electron diffraction (RHEED) with a precision better than 0.2%. After deposition, the stoichiometry is regularly checked with Rutherford back scattering (Supplementary Fig. 1).

LSMO films, deposited on STO(001) substrate or LSAT present only crystallographic phase (00l) as could be seen from XRD measurements, as shown in Supplementary Fig. 2.

The film grows fully strained up to thicknesses of 300 u.c. (120 nm) on STO and LSAT substrates; as a consequence, in-plane lattice parameters correspond to those of the substrate. For example, reciprocal space map around the asymmetric diffraction peak STO(013) of a LSMO 150 u.c. thick film deposited on STO(001) substrate, is shown in Supplementary Fig. 3. This film has the same in-plane lattice parameter as the substrate, while out-of-plane ones are different.

With STO(001) substrates the strain is tensile, while on LSAT substrate the strain is almost absent. Pseudo-cubic out-of-plane LSMO film lattice parameter depends on the substrate induced strain and in case of STO(001) substrate is equal to 0.384 nm. Good crystalline quality of LSMO films is confirmed by rocking curve measurements. LSMO films deposited on STO(001) (Supplementary Fig. 4) show a rocking curve shape on LSMO(002) diffraction peak similar to the one of the substrate while full width at half maximum (FWHM) of rocking curve has a same value as the substrate.

Due to the complex interaction between charge, orbital and magnetic orders in manganites, all of them effectively influence the double-exchange transport mechanism. As a result, the

resistivity of the film and its Curie temperature directly depend upon film quality. MBE-grown LSMO films with thickness more than 5 nm have no in-plane anisotropy in electrical and magnetic properties. Resistivity of optimally doped LSMO 100 u.c. thick film reaches the value of 70 $\mu\Omega$ cm (Supplementary Fig. 5) and Curie temperature can be estimated as 345 ± 5 K (defined as the derivative maximum), while for bulk crystal of the same doping level Curie temperature reaches 370 K and resistivity approaches 100 $\mu\Omega$ cm [2]. Squared hysteresis loops with a coercive field of about 9 Oersted were measured by magneto optical Kerr effect (MOKE).

Films have a flat surface with clearly visible (by AFM) regular terraces 0.4 nm high (up to 20 u.c. of thickness) (Supplementary Fig. 6), confirming previous reports [3-5]. RHEED pattern shows half-order diffraction peaks corresponding to oxygen octahedral around Mn atom distortion.

Supplementary Note 2: Structural and magnetic characterization of (Ga,Mn)As thin films

Ferromagnetic (Ga,Mn)As films (Mn doping level between 5% and 13%) were grown by molecular beam epitaxy using a modified Veeco Gen II system, following a well established procedure described in Ref. [6]. Thickness ranging between 20 and 300 nm were grown. Details on the nominal thickness of the samples and the exact growth temperature are given in Supplementary Table 1.

The nominal Mn concentration was determined by secondary ion mass spectroscopy (SIMS) measurements. The absolute Ga deposition rate was determined by RHEED oscillations on GaAs(001). Details on the sample growth and the used MBE system are given in ref. [6].

The magnetic properties of the samples were studied using superconducting quantum interference device (SQUID) magnetometry and by MOKE over a wide temperature range ($10 < T < 300$ K). The Curie temperature T_C measured with SQUID is obtained from the same piece of (GaMn)As measured with HAXPES. To extract T_C, from the SQUID data, the magnetization was saturated in high field and subsequently the temperature dependent magnetization *m(T)* was measured in a field of 100 Oe. T_C was taken as the inflection point of the measured *m(T)* curve. Supplementary Fig. 7 shows typical results for 6% and 12% Mn doped films with

different thickness. Post-annealing of the sample has been purposely avoided, in order to exclude MnAs cluster segregation on the surface.

Supplementary Note 3: Contamination and HAXPES

Reliability of HAXPES results are strictly connected to a careful control of contamination. Results shown in Fig. 1 and Fig. 2 display an evolution of the satellite features, while preserving a general structure of the core level spectra. Survey spectra of both LSMO and (Ga,Mn)As thin films do not display significant contamination of carbon (< 5% in all measured samples). In case of important contamination, not only limited to surface contamination, the overall structure of the spectrum changes significantly, as shown in Supplementary Fig. 8. The spectrum from the contaminated sample is from the same sample. The relative intensity of the metallic well-screened peak at low BE vs the satellite structures is severely modified. The well screened satellite appears only in the HAXPES regime. Similar results have been obtained in LSMO samples.

Supplementary Note 4: Analysis of surface roughness

Atomic Force Microscopy (AFM) analysis has been performed on both (Ga,Mn)As and LSMO samples. AFM imaging was performed on a Multimode 8 microscope equipped with a Nanoscope V controller and type J piezoelectric scanner (Bruker, USA). Samples were scanned at 0.5 Hz/line in PeakForce mode using Scanasyst-Air probes (Bruker, USA) in air, imposing an applied force of about 2.5 nN. Background interpolation and quantitative surface characterization were performed with Bruker's software or with Guiddion's software.

(GA,Mn)As

For AFM analysis, samples for the same batch were cut in two pieces. One piece was untreated; the other piece was chemically etched following the established procedure for removing spurious contamination from the surface. Samples used for HAXPES and spectroscopy characterization are etched ones. Details of chemical etching procedure are found in ref. [7]. In both cases, samples were washed by ultra-pure water, dried under nitrogen flux and measured in air within about 10 minutes. Root mean squared area roughness values were determined by averaging at least three different 3x3 μm^2 and 10 x10 μm^2 areas using the standard deviation of these measures as the uncertainty. Supplementary

Fig. 9 reports results of the untreated sample. The distribution of outgrowths (size ranging between 100 and 500 nm, thickness ranging between 3 and 12 nm) is not homogeneous. The RMS roughness is 1.05$\pm$ 0.20 nm which reduces to 0.4 $\pm$ 0.10 nm if measured in between the outgrowths.

Supplementary Fig. 1 shows AFM images of the sample after chemical etching. Note after etching the number of outgrowths (size between 100 and 200 nm with a thickness between 3 and 10 nm) were strongly reduced with respect to the untreated. The RMS roughness of etched sample is 1.51$\pm$ 0.25 nm and is less sensitive to the presence of the outgrowths. The RMS roughness measured in between the outgrowths is 1.21 $\pm$ 0.15 nm.

LSMO

Root mean squared area roughness values were determined, after AFM measurements, by averaging at least three different 5x5 μm^2 and 50 x50 μm^2 areas. Sample shows parallel grooves 15 μm spaced with some small outgrowths (< 5 nm thick) rarely present. The measured roughness for thin films with thicknesses above 40 u.c. is 0.60±0.15 nm (0.32 ±0.10 nm if measured in between the grooves). The small modulation appearing in Supplementary Fig. 11 (5x5 μm^2 image) is due to the terraces of the substrate and it does not influences the mean roughness.

Supplementary Note 5: Peak Fitting procedure

In order to estimate the contribution of the extra-peak to the total photoemission signal, a fitting procedure of the lineshapes is required: since the extra-peak in the Mn 2*p*1/2 component is not very well resolved, we restricted our analysis to the case of the Mn 2*p*3/2. The lineshape is the result of the superposition of various peaks associated to different oxidation states, which in turn are mixed by hybridisation and display a multiplet structure, which in the experimental spectra is not resolved.

We started our analysis from the spectrum acquired at 5940 eV which displays the most intense extra-peak. We succeeded in reproducing the extra-peak only by using a Gaussian. We managed to account for the rest of the lineshape by using five Gaussian lines (see Supplementary Fig. 13). We left the parameters of the peaks free when passing from a photon energy to another as reported in Supplementary Tab. 2. As detailed in Supplementary Fig. 13, Peak 1 represents the extra-peak, while Peak 2 - 6 reproduce the

rest of the Mn 2*p*3/2 lineshape. The background was fitted by using a Tougaard-type line. The standard deviation around zero of the residuals (difference between the data and the fit) was in most of the cases <0.15% and always <0.2%.

We applied a similar approach to the case of the fitting of the Mn 2*p*3/2 measured from the $La_{0.67}Sr_{0.33}MnO_3$ spectra in the metallic phase (T = 200 K). In this case when fitting the lineshapes, other than the evaluation of the integral background, a major source of uncertainty derived from the determination of the area of the satellite peak at 1000 eV, where the feature it is not resolved. As for the case of $Ga_{0.87}Mn_{0.13}As$ sample, the fitting parameters have been left free to move. The parameters obtained from the fitting procedure are shown in Supplementary Tab. 3. The standard deviation around zero of the residuals was always <0.5%.

Supplementary Note 6: X-ray penetration depth in HAXPES

The grazing incidence geometry is a relevant parameter affecting HAXPES measurements, as when the low incidence angle geometry is exploited, the x-ray penetration depth might decrease markedly to values of the same order as the photoelectron escape depth [8]. For fixed grazing incidence angle, this effect is more relevant for the soft rather than the hard x-ray photon energies. However, we show here that in our experiment the penetration depth of the incident x-ray is always larger than the Mn2*p* photoelectron escape depth by about one order of magnitude. This result comes out through comparing the attenuation length of the incident x-rays and the Mn 2*p* photoelectrons for each excitation energy adopted in our experiment. We remind here that the propagation of the incident X-rays across the solid is characterized by the attenuation length (λ_X), namely the depth into the material measured along the surface normal where the intensity of x-rays falls to 1/e of its value at the surface. Accordingly to Ref. [9], we have estimated the values of λ_X as a function of the grazing incidence angle of the x-ray beam for all the photon energies adopted in our experiment, as shown in Supplementary Fig. 14 for both materials, using the optical constants available in Ref. [10]. For the 3-degrees grazing incidence geometry, λ_X ranges from about 8-9 nm to more than 200 nm passing from 800 eV to about 6 keV, respectively. These values are compared in Supplementary Fig. 15 to the correspondent electron attenuation length λ_{e^-} of the Mn 2*p* photoelectrons calculated with the TPP-2M formula. [11,12] The kinetic energy of the Mn 2*p*

photoelectrons, assuming a Binding Energy of 640 eV, ranges from about 140 eV (for hν = 800 eV) to 5300 eV (for hν = 5940 eV), thus spanning the so-called "universal curve" of λ_{e^-} from around the minimum to the larger values of the right branch of the curve, respectively. The take-off angle of detection of the photoelectrons is about 3-degrees off the normal to the surface, hence our experimental geometry maximizes the bulk sensitivity and makes angular effects on λ_{e^-} negligible. The results of Supplementary Fig. 15 demonstrate that for the Mn 2*p* photoelectrons the condition $\lambda_X >> \lambda_{e^-}$ is largely fulfilled for all the photon energies adopted in our experiment. We can therefore conclude that the penetration depth of the X-rays is always larger than the photoelectron escape depth, thus ensuring about the consistency of our measurements over the whole interval of photon excitation energies.

Supplementary References

1. Haeni H.J, Theis C.D., Schlom D.G., RHEED Intensity Oscillations for the Stoichiometric Growth of SrTiO3 Thin Films by Reactive Molecular Beam Epitaxy, J. Electroceramics **4**, 385 (2000)
2. Urushibara A. et al. Insulator-metal transition and giant magnetoresistance in La1−xSrxMnO3, Phys. Rev. **B 51**, 14103 (1995)
3. Horiba K., et al. In vacuo photoemission study of atomically controlled La1−xSrxMnO3 thin films: Composition dependence of the electronic structure, Phys. Rev. **B 71**, 155420 (2005).
4. Feng Y. et al. Insulating phase at low temperature in ultra thin La0.8Sr0.2MnO3 films, Sci. Rep. **6**, 22382; doi: 10.1038/srep22382 (2016).
5. Konstantinovic Z., Sandiumenge F., Santiso J., Balcells L. Martıneza B., Self-assembled pit arrays as templates for the integration of Au nanocrystals in oxide surfaces, Nanoscale **5**, 1001(2013)
6. Wurstbauer U. et al. Ferromagnetic GaMnAs grown on (110) faced GaAs, Appl. Phys. Lett. **92**, 102506 (2008).
7. F. Maccherozzi et al., Influence of surface treatment on the magnetic properties of (Ga,M)As thin films, Phys. Rev. **B 74**, 104421 (2006).
8. C. S. Fadley, Progress in Surface Science, 16, 275 (1984)
9. J.D. Jackson, Classical Electrodynamics
10. X-ray database of the Center of X-Ray Optics http://henke.lbl.gov/optical_constants/

11. C. J. Powell, Surf. Sci. 44, 29 (1974); I. Lindau and W. E. Spicer, J. Electron Spectrosc. 3, 409 (1974).

12. S. Tanuma, C. J. Powell, D. R. Penn, Surf. Interf. Anal. 21, 165 (1994).
